# A Great Catch for Investigating Inborn Errors of Metabolism—Insights Obtained from Zebrafish

**DOI:** 10.3390/biom10091352

**Published:** 2020-09-22

**Authors:** Maximilian Breuer, Shunmoogum A. Patten

**Affiliations:** 1 INRS—Centre Armand-Frappier Santé et Biotechnologie, 531 Boulevard des Prairies, Laval, QC H7V 1B7, Canada; kessen.patten@iaf.inrs.ca; 2 Centre d’Excellence en Recherche sur les Maladies Orphelines–Fondation Courtois (CERMO-FC), Université du Québec à Montréal (UQAM), Montréal, QC H3C3P8, Canada

**Keywords:** zebrafish, inborn errors of metabolism, disease model, genetic disorders

## Abstract

Inborn errors of metabolism cause abnormal synthesis, recycling, or breakdown of amino acids, neurotransmitters, and other various metabolites. This aberrant homeostasis commonly causes the accumulation of toxic compounds or depletion of vital metabolites, which has detrimental consequences for the patients. Efficient and rapid intervention is often key to survival. Therefore, it requires useful animal models to understand the pathomechanisms and identify promising therapeutic drug targets. Zebrafish are an effective tool to investigate developmental mechanisms and understanding the pathophysiology of disorders. In the past decades, zebrafish have proven their efficiency for studying genetic disorders owing to the high degree of conservation between human and zebrafish genes. Subsequently, several rare inherited metabolic disorders have been successfully investigated in zebrafish revealing underlying mechanisms and identifying novel therapeutic targets, including methylmalonic acidemia, Gaucher’s disease, maple urine disorder, hyperammonemia, TRAPPC11-CDGs, and others. This review summarizes the recent impact zebrafish have made in the field of inborn errors of metabolism.

## 1. Introduction

### 1.1. Rare Inherited Metabolic Disorders

Monogenic congenital disorders, affecting a metabolic pathway, commonly resulting in a deficiency or accumulation of a specific metabolite, have been coined “inborn errors of metabolism” (IEM) [1]. This large collection of rare genetic diseases includes over 700 genetic diseases. Even though the occurrence of a specific IEM is rare to extremely rare, collectively they have an incidence rate of up to 1 in 800–5000 newborn [2,3,4]. Additionally, the occurrence and frequencies differ among regions, ethnicities, and populations [5,6,7,8,9,10]. With the advent of fast and efficient high-throughput sequencing, the number of genes associated with IEM is continuously increasing. Importantly, the use of whole-exome sequencing in the clinical setting has enhanced the diagnosis efficiency of IEM during prenatal and newborn screening. Furthermore, it has simplified the identification of mutations and genes involved in previously unresolved cases of metabolic disorders, therefore, enabling us to investigate the genes of interest, as well as genotype-phenotype correlations [11,12,13]. IEM account for a large factor of morbidity and mortality among children and who often have no available treatment or/are diagnosed with IEM at a too late stage to be treated. Some IEM have also been linked to sudden infant death [14]. Detection of IEM within the first 48 hours is thus vital to prevent any physical and/or mental retardation, yet proper diagnosis and testing of IEM are still challenging [15,16]. One effective way for clinicians to detect and combat IEM is via newborn screening, which analyzes key metabolites of newborns to identify biomarkers as early as possible and initiate treatment [17]. Treatment, if available, includes dietary control/restrictions and/or compound supplementation [18,19]. Recently additional treatment options for IEM have become available, including enzyme replacement therapy. Even though IEM are often considered as pediatric diseases, they can manifest later in life [20,21]. To advance our knowledge in the understanding of the underlying mechanisms of IEM, as well as to identify therapeutic targets, an efficient model, that can be designed successfully for the various genes and pathways, is needed. Furthermore, to improve the translatability of whole exome/genome sequencing, the decisive advantages of the models must be determined along with careful interpretation of data to accurately correlate results [22].

### 1.2. Animal Models of IEM

**Figure 1:**
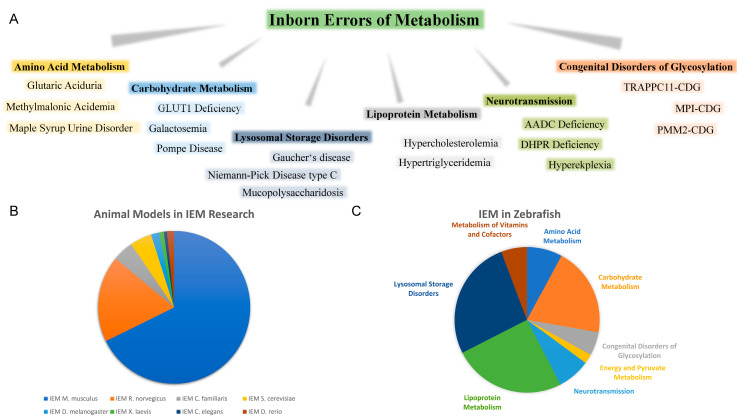
Overview of inborn errors of metabolism research. (**A**) Schematic presentation of major IEM Groups and selected disorders. (**B**) Diagram of animal models since the year 2000 used in IEM research based on Pubmed (https://www.ncbi.nlm.nih.gov/) hits (Search terms: “Inborn errors of metabolism + animal model”) last accessed 22.06.2020 (**C**) Zebrafish publications in relation to IEM studied since the year 2000 based on Pubmed research hits (Search terms based on IEM disorder group and individual disorders) last accessed 07.08.2020.

Animal models are commonly used to model and understand the underlying mechanisms of human diseases. The most commonly used animal in IEM investigations is *Mus musculus* (mouse) in about two-thirds of all studies, followed by *Rattus norvegicus* (rat) (Figure 1B). However, genotype-driven mutant generation often creates null-alleles which in the case of IEM is often a downfall, as some enzymatic activity may be retained in patients [23]. Nonetheless, numerous other model organisms are of interest to study IEM, such as *Saccharomyces cerevisiae* (yeast) [24], *Caenorhabditis elegans* (worm) [25,26,27], *Drosophila melanogaster* (fruit fly) [26,28,29], *Felis catus* (cat), and *Canis familiaris* (dog) [30,31,32], as well as *Danio rerio* (zebrafish). Among these model organisms, zebrafish only represent up 1.5% of IEM related Pubmed hits (Figure 1B). Nonetheless, zebrafish have become of special interest in the investigation of metabolism in development [33], given its numerous advantages. Of those IEM investigated in zebrafish, the majority of the studies are focused on carbohydrate metabolism, lipoprotein metabolism, and lysosomal storage disorders (Figure 1C), however, all categories of IEM have been studied. While this simple search may under- or overrepresent certain factors and models, it gives an insight into the recent selection of animal models used for IEM research. 

### 1.3. Advantages of Zebrafish for Investigating Rare Inherited Metabolic Diseases

In the last decades, zebrafish as a model organism has become an increasingly important tool in the understanding of rare inherited metabolic disorders and the development of novel therapeutic targets. The use of zebrafish has been rapidly increasing since the initial studies performed by George Streisinger [34]. In comparison to other animal models and cell culture, zebrafish have major advantages. Firstly, zebrafish have a high evolutionary conservation of genes and proteins compared to humans allowing analysis of genes associated with human disorders. Secondly, they develop rapidly enabling for first metabolic screens after 24 h post-fertilization (hpf). The simple genetic manipulation of larvae with approaches including CRISPR/Cas9, TALEN, morpholino targeting, and transgenic modification, also make this model organism a highly efficient tool for investigating inherited disorders such as IEM. Importantly, CRISPR/Cas9 mediated knockout efficiency has improved substantially in recent years with an ever-improving toolbox to generate zebrafish disease models [35,36,37,38].

Zebrafish development is incredibly well characterized regarding staging, neurological structures, cell migration/differentiation, and the zebrafish genome is also fully sequenced [39,40,41,42,43,44]. During teleost evolution, the zebrafish genome underwent a whole-genome duplication and therefore many human genes contain multiple zebrafish ortholog counterparts [45]. The resulting expression patterns of genes and orthologues may be more restrictive, allowing for investigation of protein function in development and tissue formation without resulting in embryonic lethality [46]. Furthermore, in some cases of IEM, a hypomorphic model may be more efficient to model disorders where some enzyme activity remains.

**Figure 2:**
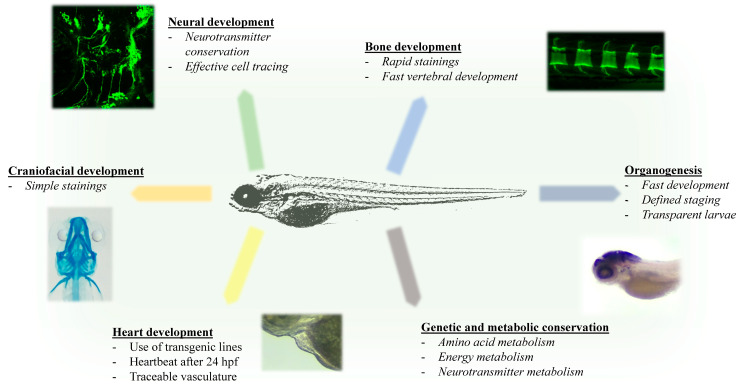
Advantages of zebrafish for metabolic studies. Overview of zebrafish advantages in regards to metabolic studies and metabolic conservation. The Figure highlights key developmental features that are important in investigating common characteristics of IEM, including heart deficits, craniofacial, spinal, as well as neural and organ abnormalities (Images presented: Neural development: lateral view of 72 hpf tg(isl:GFP) transgenic line; Bone development: close up of vertebral calcein staining of 4-week old zebrafish larvae; Craniofacial development: Dorsal view of Alcian blue staining of 6 dpf zebrafish larvae; Heart development: close up of 72 hpf zebrafish heart, Genetic and Metabolic conservation: whole-mount *in situ* hybridization of glutamine synthetase in 5 dpf larvae).

The use of zebrafish to model IEM requires highly conserved metabolic enzymes and pathways between humans and zebrafish. Zebrafish have been proven as highly effective in vivo models in this regard [47,48,49]. Recent research has also shown that metabolic profiling is fast and efficient for zebrafish larvae. Larvae can be profiled by mass spectrometry and HPLC to give a conclusive metabolic pattern of amines, amino acids, sugars, fatty acids, and citric acid cycle metabolites as early as 72 hpf [50,51]. Concurrent with the development of key metabolic organs, such as the liver, a stable profile could be determined during larval stages to investigate the metabolic role in tissue development [51]. Additionally, as many IEM results in severe neural and neurodegenerative symptoms, zebrafish are effective in modeling behavioral and neurodevelopmental diseases and their characteristics [52] (Figure 2). 

Another major advantage of zebrafish is that it is amenable to high throughput drug screening. There are increasing reports of drug screen assays and small molecule testing in zebrafish disease models and/or on behavioral neurodevelopmental aspects [53,54,55,56,57]. Interestingly, several therapeutics identified in zebrafish have started to be translated into human clinical trials [56]. Recently zebrafish have also become of great interest in clinical studies due to the development of personalized avatars and therewith developing personalized treatment approaches [58,59]. 

This review provides a comprehensive summary of the most recent advances in rare metabolic disorder studies using the zebrafish vertebrate model (summarized in Table 1). 

## 2. Amino Acid and Peptide Metabolism

One of the major groups of IEM results in the accumulation of organic acids due to a deficiency in the intermediary metabolism of amino acids, carbohydrates, and fatty acid oxidation [60,61]. Clinical features of organic acidurias, among hyperammonemia, include a wide range of symptoms such as developmental delay, mental retardation, hypoglycemia, seizures, and cardiac problems. Treatment involves a targeted restriction with additional supplementation of amino acid formulas [62]. Organic acidemias are grouped into maple syrup urine disease, methylmalonic acidemia, propionic acidemia, and isovaleric acidemia. 

### 2.1. Maple Syrup Urine Disease (MSUD) 

MSUD is a metabolic disorder resulting in branched-chain ketoaciduria, which is an accumulation of branched-chain amino acids (BCAA), such as valine, leucine, and isoleucine, and is caused by mutations in either of three genes, *BCKDHA*, *BCKDHB* or *DBT* [63]. MSUD causes developmental delay, behavioral abnormalities, and neurotoxicity. A *dbt^−/−^* zebrafish mutant, termed “quetschkommode” has been shown to effectively reproduce the metabolic profile of MSUD, with increased BCAA, depletion of glutamate in the brain, as well as abnormal swimming behavior [64]. Such CRISPR/Cas9-based knockout models mimicking disease symptoms are incredibly useful for developing specific therapeutic strategies [57,65,66,67].

Recently, zebrafish were exposed to high levels of leucine and used to mimic MSUD to investigate psychological changes as observed in patients. This study showed an altered, cholinergic related behavior in zebrafish following exposure to high levels of leucine, suggesting another efficient model to study the effect of BCAA accumulation on behavior and neurochemistry in MSUD [68].

### 2.2. Methylmalonic Acidemia (MMA)

MMA is the most common organic acidurias with an incidence rate of 1:50,000 in the United States [61]. MMA is caused by either direct or indirect effect on the conversion of methylmalonyl-CoA to succinyl-CoA by methylmalonyl-CoA mutase (*MMUT*), which is vitamin B12 dependent [69,70,71]. A mouse knockout model for MMA showed neonatal lethality [72].

Mutations in the *MMUT* gene results in toxic accumulation of methylmalonic acid, propionic acid, and 2-methylcitric acid in the mitochondrial matrix. A zebrafish CRISPR/Cas9 knockout model for *mmut* exhibited altered mitochondrial morphology in both kidney and liver of larvae, along with increased mitochondrial oxidative stress [73,74]. The high mortality in larvae could be recovered with a low protein diet, which is also the current approach for the treatment of patients. This knockout model was used to test results from a drug-disease network-based computational modeling approach to identify possible target pathways [75]. The study showed that mitochondrial stress can be targeted separately from the methylmalonic acid accumulation, by using the known drug Mito-Q, which is commonly used in lysosomal storage disorders. Treatment did ameliorate both phenotypic severity and oxidative stress, indicating the importance of targeting pathways beyond the fundamental cellular mechanisms of the disease.

Directly related to MMA is cobalamin C deficiency, which results in ineffective processing and trafficking of vitamin B12. The lack of the cofactor for MMUT, subsequently perturbates its enzyme activity. A zebrafish knockout model of the causing gene *mmachc* is the first viable model organism, displaying typical disorder symptoms including MMA, retinopathy, and juvenile lethality [76]. The model proved to be effective for testing therapeutic approaches, with findings showing that small molecules already used for patients, such as hydroxocobalamin and methylcobalamin, recover the disease severity in this model. 

### 2.3. Multiple Acyl-CoA Dehydrogenase Deficiency/Glutaric Aciduria Type II

Glutaric aciduria type II is considered as an IEM of fatty acid oxidation, but it is also directly implicated in BCAA metabolism. A zebrafish knockout mutant for the electron transfer flavoprotein dehydrogenase (*etfdh*), termed “*xavier*”, closely mimics the characteristics observed in glutaric aciduria type II [77,78]. The mutant exhibited mitochondrial dysfunction, abnormal motility, typical metabolic profiles of fatty acids, and increased neural proliferation along with cell death. This model also provided the identification of the PPARG-ERK pathway as a potential target, where specifically peroxisome proliferator-activated receptor gamma (PPARG) antagonists were found to ameliorate neural symptoms such as observed paralysis in *etfdh* zebrafish mutants. 

### 2.4. Other Amino Acid Disorders

The knockdown approach has also been used to create a zebrafish model for 3-methylglutaconic aciduria/Barth syndrome, an X-linked disorder, with organic aciduria, cardiomyopathy, and growth retardation. This model was effectively used to mimic the patient’s phenotypes and can be used to understand disease mechanisms [79]. Furthermore, in a knockdown based zebrafish model for prolidase deficiency, heart defects were validated which were previously observed in a mouse mutant [80].

## 3. Neurotransmission

Monoamine neurotransmitter disorders have been of high interest for pediatricians and scientists alike for their fundamental role in neural development and function. Studies identified tyrosine hydroxylase deficiency, tryptophan hydroxylase deficiency, and phenylalanine hydroxylase deficiency (also known as Phenylketonuria or PKU) in their role for the synthesis of fundamental neurotransmitters precursors L-DOPA, 5-HT, and amino acids, respectively. Considering this long-standing research interest, many efficient models, including mouse and cell culture, and efficient patient analysis have already been established. This holds true, especially regarding PKU. Nonetheless, researchers have investigated neurotransmitter deficiencies in zebrafish, especially for pathways surrounding the neurotransmitter synthesis and the cofactor tetrahydrobiopterin (BH_4_) pathway.

### 3.1. Aromatic L-Amino Acid Decarboxylase (AADC) Deficiency

AADC-deficiency is a neuro-metabolic disorder resulting in developmental delay, hypotonia, and dystonia, along with a depletion of biogenic amines such as dopamine and serotonin. A zebrafish model has been designed to target the gene dopa decarboxylase (*ddc*), which actively catalyzes the synthesis of dopamine and serotonin [81]. Pharmacological inhibition and knockdown based approaches of *ddc* activity in zebrafish, strikingly resulted in AADC-deficiency exhibiting clinical manifestations, including developmental delay, seizures, and abnormal swimming behavior. Notably, altering *ddc* levels in zebrafish caused abnormal dopaminergic neuronal patterning of the midbrain, which likely affects CNS function. 

### 3.2. Atypical PKU/BH_4_ Deficiency

Dihydropteridine reductase (DHPR) deficiency causes severe symptoms in children, including developmental delay, hypotonia, seizures, microcephaly, and hyperphenylalaninemia. There are highly conserved DHPR homologs in zebrafish. A recent study in zebrafish reported that two *DHPR* homologs, *qpdra,* and *qdprb1* act on separate pathways [82]. On one hand, *qdpra* was linked to the production of BH_4_ in liver and melanocytes and the zebrafish *qdpra* loss-of-function model replicates the metabolic phenotype in patients, including hyperphenylalaninemia. On the other hand, *qdprb1* is required for neural crest differentiation and proliferation without regulating BH_4_ metabolism. Zebrafish *qdprb1* knockdown resulted in small brains compared to the brain atrophy and neural symptoms in patients. It is proposed that human DHPR may function in both pathways, resulting in the very severe phenotype seen in DHPR deficiency patients. 

### 3.3. Hyperekplexia

Glycinergic synaptic transmission mediated by pentameric glycine receptors (GlyR) is essential in vertebrate CNS. Mutations that result in altered aggregation of glycine receptors cause startle disease, also known as hyperekplexia. A zebrafish mutant termed “*bandoneon*” carrying a mutation in the GlyR β-subunit (*glrbb)* effectively mirrors the human phenotype of hyperekplexia. The zebrafish “*bandoneon*” mutant has absent glycinergic neurotransmission and concurrent abnormal tactile response in form of bilateral contractions [83]. Other mutants such as one termed “*shocked*”, carrying mutations in the glycinergic transporter 1 gene (*slc6a9*), appear with similar phenotypes [84]. Detailed analysis of the “*bandoneon*” mutant has shown that all known 7 alleles act with varying sensitivity to glycine [85]. A recent study has created knockouts of all GlyR α-subunits [86]. Due to identified functional redundancy in *glr2*, *glra3*, *glra4a, glra4b* mutants showed no locomotive phenotype. However, the “*hitch*” zebrafish mutant line carrying a mutation in *glra1* displays motor dysfunction in form of reduced activity and coordination. The availability of various mutant lines in zebrafish is of great interest for future investigation of glycinergic transmission and hyperekplexia.

## 4. Metabolism of Vitamins and Co-Factors

### DHFR Deficiency

Dihydrofolate reductase (DHFR) is required for the regulation of intracellular folate homeostasis. DHFR deficiency results in megaloblastic anemia and neurological symptoms, including cerebellar atrophy, epilepsy, and retardation in patients. A zebrafish knockdown model for DHFR deficiency resulted in cardiac malformations which could be recovered by *hand2* overexpression [87]. Another approach to study *dhfr* deficiency in zebrafish is by using the specific DHFR inhibitor, methotrexate (MTX) [88,89]. Supplementation of folic acid could compensate for the effects on cardiac development in zebrafish exhibiting features of *dhfr* deficiency [90]. The knockdown of *dhfr* was also shown to regulate more fundamental pathways such as the hedgehog pathway, resulting in decreased proliferation and increased cell death [91]. A complete analysis of the folate pathway showed its role in early zebrafish development and supported the presentation of phenotypes upon *dhfr* deficiency [92]. Recently, *dhfr* transgenic lines have become available to identify expression dynamics [93]. Zebrafish *dhfr* has also been used to investigate chaperone assisted refolding [94]. Furthermore, a light-inducible drug for timed inhibition of DHFR is available and could be used for further investigations in zebrafish [95]. Using various approaches, DHFR deficiency can effectively be investigated in zebrafish.

## 5. Urea Cycle Disorders

### Hyperammonemia

Hyperammonemia is the direct consequence of urea cycle disorders. Among urea cycle disorders are ornithine transcarbamylase deficiency, arginase deficiency, and citrullinemia. The high levels of ammonia result in severe neurotoxicity, coma, and death [96]. As the enzyme deficiency results in the accumulation of a toxic compound, zebrafish can be used to mimic the acute accumulation of ammonia. A successful zebrafish model for hyperammonemia was reported, where it was shown that exposing larvae to increasing levels of ammonia in media, resulted in increasing lethality due to severe neurotoxicity [97]. Treatment with drugs commonly used in hyperammonemia to target the neurotoxicity observed in glutamatergic neurons and astrocytes showed an efficient delay in lethality. A recent study validated the model to investigate the role of acute hyperammonemia and identified a promising drug target by irreversible inhibition of ornithine aminotransferase using 5-fluoromethylornithine, efficiently recovering mortality in larvae [98]. This drug target is a promising approach to treat hyperammonemia and will likely be further investigated in the future. Follow up studies underlined the necessity for efficient intervention during hyperammonemia, by showing that prolonged hyperammonemia depleted ATP levels and resulted in bioenergetic failure [99]. Furthermore, the zebrafish model displayed decreased glutamate levels along with increased levels of gamma-aminobutyric acid (GABA), suggesting this model as a promising tool for future high-throughput drug screening [100]. 

## 6. Carbohydrate Metabolism

The body is dependent on effective energy storage. Glucose is stored as glycogen, primarily in the liver, skeletal muscle, and brain. Various types of glycogen storage disorders are caused by a deficiency in the glycogen metabolism pathways and vary from very mild to severe [101,102]. 

### 6.1. Glycogen Storage Disease Type II/Pompe Disease

Glycogen storage disease type II, also termed Pompe disease, is caused by mutations in the acid alpha-glucosidase (*GAA*) gene. The initial zebrafish model designed to disrupt *gaa* function failed to display the severity of symptoms seen in patients [103]. However, mild symptoms and excessive glycogen accumulation could be observed, which in this case could help identify underlying compensating mechanisms. However, a null mutant may be ineffective in investigating the disease phenotype, as patients often have remaining enzyme activity.

Along these lines, a very recent hypomorphic zebrafish model showed a greater resemblance to Pompe disease phenotypes such as glycogen accumulation in lysosomes and muscle stiffness. Furthermore, the study identified 3-bromopyruvic acid (3-BrPA) as a new compound ameliorating some of the observed symptoms, proposing a novel model to screen potential therapeutic interventions [104].

### 6.2. Glycogen Storage Disease Type XI/Fanconi–Bickel Disease

Fanconi–Bickel disease is caused by mutations in the gene of glucose transporter 2, *SLC2A2*, a gene that is exceptionally well conserved in zebrafish in sequence, structure, and function [105]. A knockdown model was used to analyze the role of *slc2a2* in zebrafish brain development [106]. Hypomorphic zebrafish had decreased glucose uptake and increased cell apoptosis in the brain. Specifically, neural precursor markers of glutamatergic and GABAergic neurons were decreased due to glucose deprivation. This model can be used for investigating potential therapies for Fanconi–Bickel disease.

### 6.3. Glucose-6-Phosphate Dehydrogenase (G6PD) Deficiency

Studying G6PD deficiency in mice has been challenging as the knockout model resulted in embryonic lethality [107]. This prompted an analysis in zebrafish to test for a more efficient model. The initial model using morpholinos mimicked the patients’ symptoms, which when challenged with oxidative stressors 1-naphthol, menthol, or primaquine caused severe heart edemas and hemolysis [108]. This effective readout for oxidative stress was used in a recent study with a chloroquine induced stress test, as well as a *g6pd* F0 CRISPR/Cas9 model to show an effective reduction of oxidative stress using a small compound, AG1 [109]. The hypomorphic model, together with cell culture experiments, has also been used to understand the underlying early mechanisms. The study showed that the downregulation of G6PD dysregulated the SMAD/miR200b axis required in affected key embryological pathways such as the epithelial-mesenchymal transition (EMT). E-Cadherin was sufficient to recover the observed embryological phenotype, giving G6PD a regulatory role during EMT together with SMAD/miR200b [110].

### 6.4. Galactosemia

Galactosemia is caused by galactose-1-phosphate uridylyltransferase (GALT) deficiency, resulting in the toxic accumulation of galactose metabolites which need to be countered with an appropriate diet [111]. A TALEN mediated zebrafish knockout model of *galt* [112], acts as a supplemental model to the known mouse models [113,114]. The *galt* loss-of-function zebrafish model replicated the patients’ metabolic phenotype with the accumulation of galactose-1-phosphate (Gal-1-P) upon exposure to galactose. Zebrafish not exposed to galactose developed normally. However, fish had reduced motor activity as well as reduced fertility, in line with the neurological, motor, and gonad related phenotype seen in classical galactosemia [115,116,117]. In a study using the same zebrafish KO model, there were no observed differences in UDP-galactose (UDP-Gal) or UDP-glucose (UDP-Glc) levels compared to controls, which suggests that the pathogenic cause of galactosemia is more extensive than UDP-Gal and UDP-Glc level alterations [118].

### 6.5. GLUT1 Deficiency

Abrogation of the glucose transporter 1 (*glut1*) in zebrafish by targeted knockdown results in CNS abnormalities that can be rescued by co-injection of human *GLUT1* mRNA [119]. In line with the human phenotype, hypomorphic zebrafish had decreased glucose uptake that resulted in increased cell death. The neurodegenerative effect could be ameliorated by reducing proapoptotic Bcl-2-antagonist-of-cell-death (Bad) protein levels, yet glucose transport was not improved. This model can help to understand the role of glucose metabolism in cell death in GLUT1 deficiency. 

## 7. Lipoprotein Metabolism

The proper regulation of lipoprotein metabolism is essential for lipid transport and homeostasis in the body. Lipoproteins are composed of a hydrophobic and hydrophilic portion including building blocks such as triglycerides or cholesterols. Apolipoprotein coated particles can act as ligands and/or cofactors. An effective model organism will amplify the knowledge obtained from cell culture data to multisystemic phenotypes and developmental effects. Zebrafish tools and transgenic lines are available that simplify the investigation of lipid processing, such as Oil-Red-O. Another method involves the use of fluorescently labeled lipids used to track both lipid metabolism and transport in vivo [120].

Dyslipidemias are commonly associated with the high-risk factor atherosclerosis. Zebrafish have proven a valuable model in understanding the underlying pathways and the models have been extensively reviewed [121,122]. Here, we will give an update on recent advances and reprise some highlights revealed by the zebrafish model.

### 7.1. Hypercholesterolemia

Familial hypercholesterolemia is one of the most common lipoprotein deficiencies causing atherosclerosis and is caused by mutations in the low-density lipoprotein (LDL) receptor (*LDLR*). Even though a mouse model exists that effectively mimics the patient’s phenotype [123], a zebrafish model may prove useful for efficient and rapid drug screening. An initial hypomorphic zebrafish model for *ldlr* confirmed the conservation of function, showing increased LDL-cholesterol along with liver deposits in vasculature and liver [124]. A CRISPR/Cas9 *ldlr* zebrafish mutant may however serve as a better model for high-throughput drug screening [125]. The *ldlr* zebrafish mutant displayed hypercholesterolemia which is aggravated with a short period of high cholesterol diet (HCD), as well as the sterol regulatory element-binding protein 2 (Srebp-2) pathway activation. Furthermore, treatment of these mutants with an inhibitor of apolipoprotein B (ApoB) secretion showed reduced lipid accumulation. 

Induction of hypercholesteremia in zebrafish by feeding with an HCD can be used to investigate both their effects as well as possible treatment approaches. HCD induces endothelial inflammation along with peroxisome proliferator-activated receptor-gamma (*pparγ*) downregulation prior to lipid accumulation and atherosclerosis. Treatment with an agonist to PPARγ ameliorated the phenotype [126]. A study using berbamine identified its anti-hypercholesterolemic and hepatoprotective effect [127]. The hypercholesteremia model has also been used to identify novel antihyperlipidemic compounds, such as ginsenosides as a potential clinical tactic [128]. Finally, zebrafish, in addition to providing insights to novel therapeutic approaches, can also be used to understand the adverse effects of drugs already in use, such as statins, that are the major drug prescribed for hyperlipidemias [129].

### 7.2. Hypertriglyceridemia

A deficiency in the apolipoprotein C2 (APOC2) can be causative of hypertriglyceridemia. An *apoc2* zebrafish mutant developed severe hypertriglyceridemia, chylomicronemia, and reduced lipase activity all in line with human patients [130]. Furthermore, overgrowth of the pancreas, as well as lipid accumulation in the vasculature indicating atherosclerosis, make this model a successful representative for future investigations. 

## 8. Congenital Disorders of Glycosylation (CDG)

### 8.1. Pmm2-CDG

CDG are disorders involving an underlying deficiency in the modification of proteins by sugar moieties. Mutations in phosphomannomutase 2 (*PMM2*), the most common underlying genetic cause [131], have been previously investigated in numerous model organisms including *C. elegans* [132], *X. laevis* [133], and *S. cerevisiae* [134]. PMM2-CDG constitutes for roughly 80% of all CDG cases and currently has no successful therapeutic intervention. A zebrafish knockdown model validated the role of *pmm2* in the early neural and craniofacial development in zebrafish and was consistent with symptoms seen in CDG patients [135]. A zebrafish model [136], with a hypomorphic *pmm2* mutation, was recently developed and it successfully replicated aspects of the disease. This model identified underlying endoplasmic reticulum (ER) stress resulting in the activation of nuclear factor erythroid 2-related factor 2 (Nrf2), mediated by the pERK pathway. While the study with this model did not identify a novel drug target, it confirmed that the underlying mechanisms in PMM2-CDG are more extensive than what is currently known about the involved pathways and may open novel treatment approaches. 

### 8.2. MPI-CDG

A morpholino based study focusing on the deficiency of phosphomannose isomerase (*mpi*), in zebrafish indicated the important role of early intervention [137]. As the mouse model is embryonic lethal [138], the knockdown approach in zebrafish was effectively used to study MPI-CDG. The phenotypes in this MPI-CDG zebrafish model included small eyes, pericardial edema, and small liver. In line with MPI-CDG being the only treatable CDG, mannose supplementation was sufficient to restore glycosylation levels and rescue the phenotype observed in the morphants. Notably, the supplementation was only effective if given within the first 24 hpf.

### 8.3. PGM3-CDG

A study combining *X. laevis*, *D. melanogaster* and *D. rerio* provided a more fundamental developmental defect in correlation to UDP-GlcNAc, the building blocks of N-glycosylation [139]. The experiments showed that interfering with the UDP-GLcNAc glycosylation salvage pathway results in defective Wnt signaling. The abnormal UDP-GLcNAc pool may be in line with the mutations in phosphoglucomutase (PGM3) and phenotypes of PGM3-CDG patients [140,141].

### 8.4. TMEM165-CDG

Another study focusing on CDG showed the role of transmembrane protein 165 (*tmem165*) in the development of zebrafish craniofacial cartilage and osteoblast differentiation [142]. These results are in line with observed osteopenia or osteoporosis in TMEM-165-CDG patients [143]. The experiments particularly showed that both craniofacial abnormalities and N-glycosylation deficiency were effectively rescued by co-injection of zebrafish wildtype *tmem165* mRNA. However, the phenotype could not be recovered with zebrafish *tmem165* mRNA carrying a conserved patient mutation. This study established zebrafish as an effective model for TMEM165-CDG and for investigating the underlying mechanisms. 

### 8.5. TRAPPC11-CDG 

A genetic screen of 297 zebrafish lines identified the mutant with a gene-trap cassette inserted into the novel gene, originally termed “*foie gras*”, as an effective model for liver disease, presenting with hepatomegaly, steatosis, and increased hepatocyte death [144]. Further studies linked the model to unfolded protein response (UPR) activation and ER stress. Close similarity to tunicamycin treated zebrafish resulted in the first speculation towards glycosylation [145]. The gene “*foie gras*” was identified as an ortholog of the transport protein particle complex 11 (TRAPPC11) and patients were initially linked to limb-girdle muscular dystrophy and steatosis [146]. Following investigations using a zebrafish mutant identified a striking defect in protein glycosylation, specifically the reduction of lipid-linked-oligosaccharides (LLO) [147]. The effect on UPR was mimicked by inhibiting LLO synthesis, placing the glycosylation defect upstream of UPR. Due to the studies performed in zebrafish, TRAPPC11 mutations have been validated as one of the underlying genetic causes in CDG patients [148,149].

## 9. Energy and Pyruvate Metabolism

### Pyruvate Dehydrogenase (PDH) Complex 

PDH complex deficiencies are often severe with poor prognosis. Symptoms include developmental delay, abnormal brain development, encephalopathy, epilepsy, peripheral neuropathy, and congenital lactic acidosis. Genetic mutations underlying the disorder have been found in *PDH1* (E1 subunit), dihydrolipoamide acetyltransferase (*DLAT*) (E2 subunit), and dihydrolipoamide dehydrogenase (*DLD*) (E3 subunit). An effective animal model is of great value in the investigation of treatments and their effectiveness. A first zebrafish model was identified and termed “*no optokinetic response a”* mutant, which carries a mutation in the E2 subunit [150,151]. In line with the diagnostic indicators, mutant fish present with increased lactate and pyruvate. The lactic acidosis could be countered successfully with a ketogenic diet in fish, improving both neurological aspects and survival. A ketogenic diet is also the suggested intervention for patients affected by PDH complex deficiency [152]. Retracting the ketogenic diet from zebrafish resulted in rapidly occurring lethality [150]. Another mutant termed “*noir*” contains a mutation in *pdh1b*, the E1 β subunit of the PDH complex [153]. The mutant displayed loss of vision, a lower number of cholinergic amacrine cells in the retina, and no response to light as screened by ERG at 7 dpf. Similar to the E2 mutant, a ketogenic diet ameliorated the severity and improved survival of the mutant. Both mutants provided successful underlining of the importance of early and consistent intervention with a ketogenic diet and will be an incredibly useful tool in future testing for ketogenic compounds or drugs. In fact, phenylbutyrate has been identified to regulate PDH complex activity by inhibiting pyruvate dehydrogenase kinase and increasing levels of activated PDH complex by phosphorylation of the E1 subunit [154]. Treatment of the “*no optokinetic response a*” mutant with phenylbutyrate, showed correction of locomotor and biochemical alterations.

## 10. Lysosomal Storage Disorders

Lysosomal storage disorders present one of the largest clusters of IEM, covering over 70 genetic deficiencies [155]. While each disease by itself is considered rare to extremely rare, taken together lysosomal storage disorders have a frequency of 1 in 5000–7700 newborns [156,157]. Many of these disorders have been investigated in zebrafish due to the high evolutionary conservation of genes and proteins between zebrafish and humans. Furthermore, the model has particular advantages such as simple stainings and compound tracking, such as lysotracker, as well as specific transgenic lines that have been generated to track lysosomal processing [157,158].

Lysosomal storage disorders including types of neuronal ceroid lipofuscinoses (NCL), sphingolipidoses, and mucopolysaccharidosis (MPS) in the zebrafish model have just recently been extensively reviewed, therefore we will only highlight some of the most recent and striking advances [159].

### 10.1. Gaucher’s Disease

Gaucher’s disease is caused by mutations in the glucocerebrosidase gene 1 (*GBA1*) and is the most common lysosomal storage disorder with an incidence rate of 1:40,000 [160]. The deficiency results in the accumulation of glucocerebrosides in lysosomes causing splenomegaly, blood disorders, and skeletal disorders. Gaucher’s disease is categorized into three types: type I (no neurological symptoms), type II (early-onset neurodegeneration), and type III (late-onset neurological symptoms) [161]. Even with type I presenting without major neurological symptoms the occurrence of Parkinson’s disease is increased by 26-fold in Gaucher’s disease patients [162]. A TALEN loss-of-function zebrafish model was the first vertebrate model to effectively mirrors the Gaucher’s disease phenotype in both visceral and neural tissues, making it an excellent model for drug screening [163]. Strikingly in this study, loss of dopaminergic neurons in the brain could be determined as alpha-synuclein independent, as zebrafish lack the human alpha-synuclein counterpart [163,164]. Neurodegeneration was likely associated with non-alpha-synuclein proteinopathy preceded by microglial activation, ‘Gaucher like cell’ invasion, and early increase in miR-155 expression [163]. A more recent study has investigated the involvement of inflammation marker miR-155, in the zebrafish model [165]. The study was able to validate the increase in miR-155 in mammalian models of Gaucher’s disease. Furthermore, a *gba1^−/−^*/*miR-155^−/−^* double mutant did neither rescue the inflammation nor disease progression, concluding that miR-155 is not a promising therapeutic target. Another study using both mutants and a knockdown approach investigated the effect of *gba1* deficiency on bone formation. The results showed a reduction in osteoblast differentiation markers and a negative regulation of the canonical Wnt pathway. Therewith, it proposed an intriguing role of the Wnt pathway in the skeletal abnormalities in Gaucher’s disease [166]. Recent studies have used the efficiency of the zebrafish to further develop efficient models and understand the underlying mechanisms. Chemical studies have developed novel specific *gba1* inhibitors based on cyclophellitol [167]. Another study has also used zebrafish to investigate the role of the *gba1* homolog *gba2* in a CRISPR/Cas9 model [168].

### 10.2. Niemann-Pick Disease C (NPC)

NPC is characterized by the accumulation of glycolipids and cholesterol in lysosomes, mediated by a deficiency in NPC1 (95% of cases) or NPC2 required for proper intracellular cholesterol trafficking [169,170,171]. Patients with NPC present with neonatal jaundice, liver disease up to liver failure, ataxia, seizures, and progressing neurological problems [172,173,174].

In a CRISPR/Cas9 mediated knockout model for NPC, *npc1*^−/−^ zebrafish mutants had increased lethality prior to 6 months of age and striking growth retardation [175,176]. Homozygous mutants presented commonly with larger, vacuole-like structures and a strong signal for unesterified cholesterol, a key hallmark of NPC. Increased unesterified cholesterol was also previously shown in a knockdown model for NPC [177]. Another knockdown model also showed hematological defects including thrombocytopenia and anemia [178]. Furthermore, *npc1*^−/−^ mutants present with similar neurological abnormalities as patients, including axonal spheroids and disorganized Purkinje cells [175,176,179]. The knockout model may serve as an effective drug-screen model, as LysoTracker Red staining (for acidic organelles) and filipin staining (for unesterified cholesterol) are increased in early development. Drug screens with 2-hydroxypropyl-β-cyclodextrin (2HPβCD), a compound confirmed in other models and tested in clinical trials, reduced staining in *npc1*^−/−^ mutants confirming the efficacy of the model [175,180,181].

### 10.3. Farber Disease

Farber disease is caused by loss of function in acid ceramidase, ASAH1, resulting in the accumulation of ceramide, causing a multisystemic phenotype associated with early mortality [182]. Deficiencies in this enzyme have also been associated with the development of spinal muscular atrophy with progressive myoclonic epilepsy (SMA-PME) [183].

A first zebrafish knockdown model showed a loss of motor neuron axonal branching [184]. A more recent study designed a CRISPR/Cas9 knockout for both orthologues *asah1a* and *asah1b*, successfully mimicking the characteristics of Farber disease [185]. Using parallel reaction monitoring (PRM)-based liquid chromatography–mass spectrometry (LC–MS) method for ceramide analysis, it was shown that a double knockout is required to significantly increase ceramide content in the zebrafish brain, as either *asah1a* or *asah1b* was sufficient to maintain physiological levels. Detailed analysis of the double knockout mutant revealed a clear trend towards the accumulation of ceramides with a higher number of carbons in the long-chain base and less in the acyl-chains of the sphingolipids, indicating a possibility for more complex compensatory mechanisms. 

### 10.4. Mucopolysaccharidosis (MPS)

MPS is a lysosomal storage disorder resulting in the accumulation of glycosaminoglycans (GAGs). Seven different types have been characterized, connected to 11 enzymes [186]. Symptoms vary widely from neurological symptoms to skeletal abnormalities [187]. Currently, treatment options focus on enzyme replacement therapy, but with the advances in gene editing and efficient zebrafish models, other treatment strategies may arise [188].

### 10.5. Mucopolysaccharidosis II (MPS II)/Hunter Syndrome

MPS II or Hunter syndrome is an X-linked condition that is caused by a deficiency in the iduronate-2-sulfatase (IDS) enzyme. Patients present with several different symptoms ranging from respiratory tract dysfunctions to a collection of bone abnormalities termed dysostosis multiplex. [187]. An initial zebrafish knockdown model identified an increased Tgfβ signaling associated with *ids* knockdown, affecting both craniofacial cartilage development and neural crest cells [189]. The same model was used to find an underlying Shh signaling downregulation and in turn Wnt/β-Catenin upregulation, during heart development before the onset of GAG accumulation [190].

A new study in zebrafish using a CRISPR/Cas9 knockout, revealed deficient Fgf signaling in 2 dpf zebrafish, that persists in adult bone tissue [191]. Key skeletal markers for mineralization, including *osx* and *bglap* were significantly downregulated and adult zebrafish presented with skeletal abnormalities revealed by microCT analysis, showing that the signaling defects appear long before the skeletal symptom onset. Additionally, the study was able to validate the downregulated FGF targets (*dups6* and *pea3*) in fibroblasts from Hunter syndrome patients. These studies showed that the underlying pathophysiology is not only caused by GAG accumulation, but by several misregulated pathways. 

## 11. Novel Therapeutic Intervention for Metabolic Disorders

With one of the greatest advantages of zebrafish being high throughput drug screening capability, it is to be expected that the listed models will be used to uncover novel therapeutic interventions. Therapeutic interventions for metabolic disorders are often simple and at the same time inefficient. While IEM can result in metabolite deficiency, supplementation appears to be the subsequent approach. However, delivery is not always efficient, especially if crossing the blood-brain-barrier is required. Possible novel targets, as well as previously unknown underlying mechanisms, as shown in this review, have been identified using zebrafish models for disorders underlying hyperammonemia, hypercholesterolemia, PDH complex deficiency, and TRAPPC11C-CDG.

## 12. Outlook

The impact made on IEM by the zebrafish model is evident. Even more, the influence zebrafish studies had in the last decades in a great variety of diseases and their relevance for human treatment has been highlighted in numerous publications [159,192,193,194,195,196,197]. It is to be expected that the role of zebrafish will continue to grow for rare metabolic disorders, especially with the prospect of drug screening and the effectiveness of CRISPR/Cas9 editing. Furthermore, with the increasing number of novel mutations identified for underlying metabolic disorders by whole-exome/genome sequencing, the simplicity of the zebrafish model to easily validate the pathogenicity of genetic mutations and elucidate their roles in disease pathogenesis will likely encourage further cooperation between clinicians and scientists in the rare metabolic disorders field. The past has already shown that such collaboration can lead to the successful investigation of gene function up to therapeutic intervention. This will increase our knowledge not only on known metabolic functions, but likely identify novel pathways, previously uncharacterized. As the large variety of rare inherited metabolic disorders has sparked great interest in research, zebrafish will likely be key in testing novel approaches for treatment, including gene therapy. In an important note: IEM are often considered only pediatric diseases, but with improving diagnosis and treatment options, adult and long-term management will become a major focus of new studies, to improve not only mutation detection but also the quality of life. 

Future studies will continue to use zebrafish and other model organisms for rare inherited metabolic disorders and play their part in contributing to novel drug targets, mechanisms, and treatment options.

## Figures and Tables

**Table 1:** Summary of Zebrafish “inborn errors of metabolism” (IEM) models.

Metabolism	Disorders	Zebrafish Genes	Approach	Reference
**Amino acid and peptide metabolism**	Maple syrup urine disorder	*dbt*	CRISPR/Cas9; Metabolite exposure	[64,68]
	Methylmalonic acidemia	*mmut, mmachc*	CRISPR/Cas9	[73,74,75,76]
	Glutaric aciduria type II	*etfdh*	CRISPR/Cas9	[77,78]
	Barth Syndrome	*taz*	Morpholino	[79]
	Prolidase deficiency	*pepd*	Morpholino	[80]
**Neurotransmission**	AADC deficiency	*ddc*	Inhibitor, Morpholino	[81]
	DHPR deficiency	*qdpra/qdprb1/qdprb2*	Morpholino	[82]
	Hyperekplexia	*glra1, glrbb*	ENU	[83,84,85,86]
**Metabolism of Vitamins and Co-factors**	DHFR deficiency	*dhfr*	Inhibitor, Morpholino	[87,88,89,90,91,92]
**Urea Cycle Disorders**	Hyperammonemia	*/*	Metabolite exposure	[97,98,99,100]
**Carbohydrate Metabolism**	GSD Type II/Pompe disease	*gaa*	Morpholino	[103,104]
	GSD type XI/Fanconi-Bickel disease	*slc2a2*	Morpholino	[106]
	G6PD deficiency	*g6pd*	CRISPR/Cas9; Morpholino	[108,109,110]
	Galactosemia	*galt*	TALEN	[112,118]
	GLUT1 deficiency	*glut1*	Morpholino	[119]
**Lipoprotein Metabolism**	Hypercholesterolemia	*ldlr*	CRISPR/Cas9, Morpholino, Diet	[124,125,126,127,128]
	Hypertriglyceridemia	*apoc2*	CRISPR/Cas9	[130]
**Congenital disorders of glycosylation (CDG)**	PMM2-CDG	*pmm2*	ENU, Morpholino	[135,136]
	MPI-CDG	*mpi*	Morpholino	[137]
	TMEM165-CDG	*tmem165*	Morpholino	[142]
	TRAPPC11-CDG	*trappc11c*	gene-trap cassette	[144,145,146,147]
**Energy and Pyruvate Metabolism**	Pyruvate dehydrogenase complex deficiency	*dlat, pdh1b*	ENU	[150,151,153,154]
**Lysosomal Storage Disorders**	Gaucher’s disease	*gba1*	TALEN; CRISPR/Cas9; Morpholino	[163,165,166,167,168]
	Niemann–Pick disease C	*npc1*	CRISPR/Cas9; Morpholino	[175,176,177,178]
	Farber disease	*asah1a, asah1b*	CRISPR/Cas9; Morpholino	[184,185]
	MPS II/Hunter Syndrome	*ids*	CRISPR/Cas9; Morpholino	[189,190,191]
